# Maternal factors associated with hyperglycemia in pregnancy and perinatal outcomes: a Brazilian reference center cohort study

**DOI:** 10.1186/s13098-020-00556-w

**Published:** 2020-06-06

**Authors:** Bianca F. Nicolosi, Joice M. Vernini, Roberto A. Costa, Claudia G. Magalhães, Marilza V. C. Rudge, José E. Corrente, Jose G. Cecatti, Iracema M. P. Calderon

**Affiliations:** 1 Graduate Program of Gynecology, Obstetrics and Mastology, Botucatu Medical School, Unesp, Botucatu, SP Brazil; 2 Department of Obstetrics and Gynecology, Botucatu Medical School, Unesp, Botucatu, SP Brazil; 3 Department of Biostatistics, Botucatu Bioscience Institute (BBI), Unesp, Botucatu, SP Brazil; 4 Department of Obstetrics and Gynecology, University of Campinas (UNICAMP), School of Medical Sciences, Campinas, SP Brazil

**Keywords:** Hyperglycemia, Gestational diabetes, Pregnancy, Perinatal outcomes, Risk

## Abstract

**Background:**

While sufficient evidence supporting universal screening is not available, it is justifiable to look for specific risk factors for gestational diabetes mellitus (GDM) or hyperglycemia in pregnancy (HIP). The objective of this study is to identify independent risk factors for HIP and its adverse perinatal outcomes in a Brazilian public referral center.

**Methods:**

We included 569 singleton pregnant women who were split into three groups by glucose status: GDM (n = 207), mild gestational hyperglycemia (MGH; n = 133), and control (n = 229). Women who used corticosteroids or had a history of DM were excluded. HIP comprised both GDM and MGH, diagnosed by a 100 g- or 75 g-oral glucose tolerance test (OGTT) and a glucose profile at 24–28 weeks. Maternal characteristics were tested for their ability to predict HIP and its outcomes. Bivariate analysis (RR; 95% CI) was used to identify potential associations. Logistic regression (RR_adj_; 95% CI) was used to confirm the independent risk factors for HIP and its perinatal outcomes (*p* < 0.05).

**Results:**

Age ≥ 25 years [1.83, 1.12–2.99], prepregnancy BMI ≥ 25 kg/m^2^ [2.88, 1.89–4.39], family history of DM [2.12, 1.42–3.17] and multiparity [2.07, 1.27–3.37] were independent risk factors for HIP. Family history of DM [169, 1.16–2.16] and hypertension [2.00, 1.36–2.98] were independent risk factors for C-section. HbA1c ≥ 6.0% at birth was an independent risk factor for LGA [1.99, 1.05–3.80], macrosomia [2.43, 1.27–4.63], and birthweight Z-score > 2.0 [4.17, 1.57–11.10].

**Conclusions:**

MGH presents adverse pregnancy outcomes similar to those observed in the GDM group but distinct from those observed in the control (no diabetes) group. In our cohort, age ≥ 25 years, prepregnancy BMI ≥ 25 kg/m^2^, family history of DM, and multiparity were independent risk factors for HIP, supporting the use of selective screening for this condition. These results should be validated in populations with similar characteristics in Brazil or other low- and middle-income countries.

## Background

Diabetes in pregnancy (DIP) and gestational diabetes mellitus (GDM), both of which are characterized by glucose intolerance first diagnosed in pregnancy, constitute a unique condition now named hyperglycemia in pregnancy (HIP). DIP is hyperglycemia diagnosed in early pregnancy using WHO diagnostic criteria for nonpregnant women; GDM is diagnosed in the second and third trimesters of pregnancy using IADPSG criteria based on the risk of adverse perinatal results [1–3]. GDM is the most common metabolic disorder that occurs during pregnancy, and it is associated with adverse short- and long-term effects on both the mother and offspring and an increased risk for future type 2 diabetes mellitus (T2DM), metabolic syndrome (MS) and cardiovascular disease (CVD) [4–7].

Milder forms of hyperglycemia that do not meet the diagnostic criteria for GDM but have adverse effects on the mother and offspring have been identified [8]. Over three decades ago, our public referral center found an association between the glucose profile (GP) test and the oral glucose tolerance test (OGTT) for the diagnosis of GDM. Regardless of normal OGTT, the abnormal GP test indicated hyperglycemia in some pregnant women; when hyperglycemia was untreated, the perinatal mortality rate was 4.16%, which is similar to the rate observed in the GDM group and ten times greater than that observed in the nondiabetic control group. These cases are subjected to strict glucose control, similar to diabetes in pregnant women, and are classified as mild gestational hyperglycemia (MGH) [9, 10].

In our referral center, the IADPSG diagnostic protocol was adopted in August 2011, but the GP test was maintained in parallel with the 75 g-OGTT. Even after this adoption, the prevalence of MGH was 17.3%, and it was associated with LGA, macrosomia, first C-section, and hospital stay above 3 days [1, 11].

Brazil is one of the eight countries that accounts for 55% of the deliveries and 55% of the diabetes cases worldwide [3]. According to the Brazilian GDM diagnostic consensus, universal screening using FPG and 75-g OGTT must be applied in settings with technical and financial resources to identify 100% of cases; in settings with nonideal conditions, fasting plasma glucose (FPG) at the first prenatal visit may be used to screen for approximately 86% of GDM cases, and if the FPG levels are normal (< 92 mg/dL) at the first prenatal visit, they should be reassessed at 24–28 weeks [12].

Although recent studies highlight a positive linear association between hyperglycemia in diagnostic tests and adverse outcomes for all glucose level exposures, there is no clear evidence regarding diagnostic tests or threshold effects. In low-income countries, the technical and financial conditions are limited, and it is important to investigate risk factors for GDM as an alternative to universal screening.

While better evidence supporting universal screening is not available and risk-based screening remains controversial and dependent on the specific characteristics of the population evaluated, it is justifiable to look for specific risk factors for GDM or other forms of hyperglycemia [13–17]. Therefore, the objectives of this study are (i) to compare the maternal and pregnancy characteristics and perinatal outcomes of women with different glucose statuses and (ii) to identify the independent risk factors for HIP and its respective adverse perinatal outcomes in a public tertiary referral center in Brazil.

## Methods

### Study design and patients

This is a cohort study that includes some previously published data [11]. The study was developed at the Botucatu Medical School/Unesp, a Brazilian obstetrical referral center. The original cohort from our Perinatal Diabetes Research Center (PDRC), with data prospectively collected and electronically stored at each prenatal visit and each event (diagnostic test for DMG or MGH, delivery, abortion, puerperium and, eventually, death), constituted the study database. Pairs of mothers and newborns assisted at our center between 1 January 2008 and 31 December 2014 were included. The inclusion criteria were as follows: underwent the diagnostic protocol for hyperglycemia in pregnancy [9, 10]; had birth assistance at our referral center. The exclusion criteria were as follows: cases of multiple pregnancies; long-term use of corticosteroids; and a history of diabetes mellitus (overt DM, T1DM or T2DM).

### Simple size

The sample size was calculated based on the frequency of maternal hyperglycemia (15–20%) and LGA newborns (12–14%) observed in our previous studies [10, 11]. Assuming a type II error of 20%, a confidence level of 95%, and 12% the minimum frequency, the desired sample size was estimated to be 554 pregnant women, including 277 without and 277 with hyperglycemia. We recruited 569 patients, including 229 control patients and 340 HIP (GDM and MGH) patients.

### Diagnosis of hyperglycemia in pregnancy

Prior to August 2011, the standard GDM diagnostic test was 100 g-OGTT—fasting plasma glucose (FPG) below 5.3 mmol/L (95 mg/dL), 1 h postload plasma glucose less than 10.0 mmol/L (180 mg/dL), 2 h postload plasma glucose less than 8.6 mmol/L (155 mg/dL), and 3 h postload plasma glucose less than 7.8 mmol/L (140 mg/dL). GDM was confirmed when 2 values were equal to or above these limits [18]. After August 2011, the 75 g-OGTT was adopted—FPG between 5.1 and 6.9 mmol/L (92–125 mg/dL) or 1 h postload plasma glucose equal to or above 10.0 mmol/L (180 mg/dL) or 2 h postload plasma glucose between 8.5 and 11.0 mmol/L (153–199 mg/dL) [1, 2, 19].

The criteria for MGH diagnosis were normal 100 g- or 75 g-OGTT and altered GP test, that is, fasting plasma glucose equal to or above 90 mg/dL (5.0 mmol/L) or 2 h postprandial plasma glucose equal to or above 130 mg/dL (7.2 mmol/L). The GP test was performed over a one-day hospital stay with the women on a 2840 kcal diet fractionated in five meals. Plasma glucose measurements were taken every 2 h between 8 a.m. and 6 p.m. [9, 10].

### Patient follow-up

All patients included in this cohort started prenatal care before 20 weeks of gestation and underwent a hyperglycemia screening protocol between 24 and 28 gestational weeks [9, 10, 18, 19].

The pregnant women with normal OGTT and normal GP were classified as nondiabetic, had prenatal care in the public health system (primary level), and had childbirth assistance at the secondary or tertiary level (our referral center). Nondiabetic women who received birth assistance at our referral center and who agreed to participate in the study were included in the control group.

Immediately after the diagnosis, both MGH and GDM pregnant women were cared for the referral center by a multiprofessional team and underwent maternal glucose control, according to the ADA’s recommendation. Lifestyle changes (diet and exercise) were first recommended, and this treatment was complemented by insulin therapy when glycemic goals were not achieved [20, 21]. Oral hypoglycemic drugs are not recommended by ANVISA (the Brazilian Health Surveillance Agency) to be used in pregnancy, so they are not prescribed in our center.

The glucose control in pregnant women with GDM and MGH was monitored every 1 or 2 weeks by the GP test and was performed with an individual-specific diet. The respective glycemic mean (GM) ≥ 120 mg/dL was defined as inadequate glucose control [9, 10].

### Maternal characteristics and perinatal outcomes

The maternal baseline characteristics were defined by maternal age at enrollment, parity, number of prenatal visits, some indicators of socioeconomic status and habits, family history of DM (first degree) and prepregnancy hypertension [18, 19]. Exercise was defined as the execution of planned, structured and repetitive corporal movements designed to improve one or more components of physical fitness, such as swimming, running and walking at an accelerated pace. The prepregnancy body mass index (BMI; kg/m^2^) was calculated using the self-reported prepregnancy maternal weight; the pregnancy weight gain (kg) was calculated by the difference between final pregnancy weight and prepregnancy maternal weight and was classified according to the prepregnancy BMI [22]. The glucose status was evaluated at the GDM screening (24–28 weeks) by 100 g- or 75 g-OGTT combined with the GP test [1, 2, 9, 10, 18, 19]. Glycated hemoglobin (HbA1c) was evaluated later in pregnancy (36–38 weeks) or at birth in nondiabetic women. HbA1c levels ≥ 6.0 or ≥ 6.5% were defined as markers for inadequate glucose control status and an increased risk of adverse perinatal outcomes [23].

The perinatal outcomes evaluated in this study were defined by the potential hyperglycemia influence and a 12–15% rate of missed data. At birth, the mode of delivery, gestational age (GA) and birthweight (BW) according to GA were assessed. The cephalic/abdominal perimeter ratio, ponderal index [PI = BW(g)/height(cm)^3^], BW/GA Z-score, and respective cutoff values complemented the evaluation of excessive fetal growth [24, 25]. Newborn sex (male or female), Apgar scores, biochemical parameters of the umbilical cord blood, malformations, and some indicators of the neonatal period, including death until 28 days and hospital length of stay, were also considered. All reference parameters were used in accordance with the clinical protocol of the local perinatal unit.

### Data collection and statistical analysis

For the current analysis, we used the database of the cohort study from our referral center [11]. According to the predefined period and the inclusion and exclusion criteria, data were input in a specific Excel software spreadsheet, audited, and underwent consistency checking. To compare the maternal and pregnancy characteristics and perinatal outcomes among the three different glucose status groups, namely, nondiabetes (ND), MGH and GDM, the Chi squared test or Fisher’s exact test was used as appropriate. To evaluate the association of HIP with maternal and pregnancy characteristics and with adverse perinatal outcomes, two study groups were defined, namely, the HIP group (MGH and GDM) and the control group (Non-HIP), and the relative risks (RR) and 95% confidence intervals (CIs) were calculated in the bivariate analysis.

Finally, logistic regression analysis was performed with the adjusted RR (RR_adj_) and 95% CI to identify the independent risk factors. In the forward model, all significant maternal and pregnancy characteristics were used as independent predictors; HIP and significant perinatal outcomes were included as outcomes. For all tests, the statistical significance limit was *p* < 0.05.

## Results

**Fig. 1:**
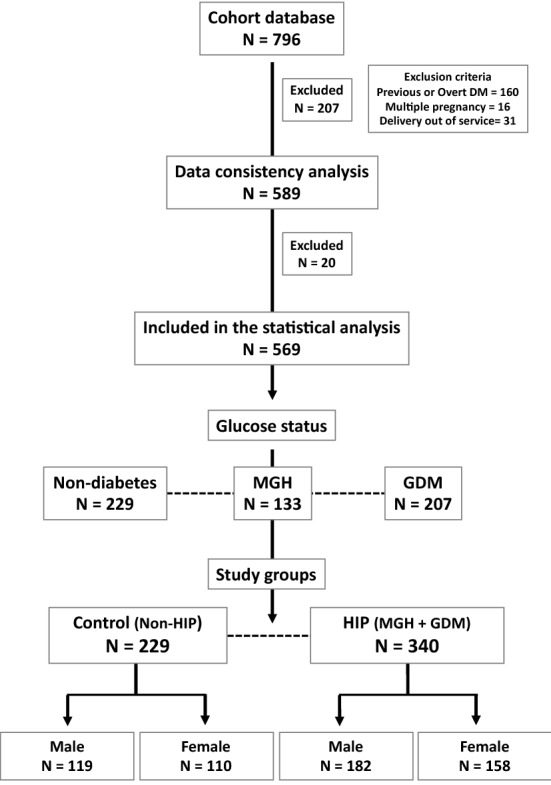
Study flowchart

The study flowchart is shown in Fig. 1. A total of 796 pregnant women were assessed for eligibility; of these, 589 were selected for data consistency checking, and 20 of those women were excluded due to data inconsistency. Thus, 569 pregnant women, with their respective newborns, were classified according to their glucose status—nondiabetes (N = 229), MGH (N = 133) and GDM (N = 207)—and then distributed into either the HIP group (MGH and GDM; N = 340) or the control group (non-HIP; N = 229). The prevalence of GDM (39.6 vs 32.2%; *p* = 0.095), HIP (GDM + MGH) (58.2 vs 62.3%; *p* = 0.1215), and non-HIP (control) (41.7 vs 37.7%; *p* = 0.3931) was not influenced by diagnostic tests, respectively, 75 g- or 100 g-OGTT.

Tables 1 and 2 show maternal and pregnancy characteristics and perinatal outcomes according to glucose status. Maternal age ≥ 25 years, multiparity (≥ 2), nonexercise/sedentarism, and family history of DM differentiated GDM and MGH from the control group but not GDM from the MGH group. The number of prenatal visits < 6 and the presence of hypertension adequately distinguished GDM from the control group; in the MGH group, these results were intermediate and similar to both the GDM and control groups. Women without a partner were less prevalent in the GDM group, and the prevalence of prepregnancy BMI ≥ 25 kg/m^2^ was proportional to maternal glucose status. Independent of 100 g- or 75 g-OGTT, the mean glucose values of OGTT and of GP were able to differentiate the GDM group from the control group; the MGH group had results that were statistically similar to the control group. Relative to glucose control in pregnancy, cutoff HbA1c levels of ≥ 6.5 or 6.0% were both adequate to distinguish hyperglycemia (GDM and MGH) from the control group (Table 1).

**Table 1:** Maternal and pregnancy characteristics according to glucose status

Characteristics	GDM	MGH	ND	*p* value*
	N (%)	N (%)	N (%)	
Age ≥ 25 years	181 (87.86)a	108 (81.20)a	143 (62.72)b	*<0.0001*
Prenatal visits < 6	24 (13.04)a	10 (9.17)ab	11 (5.26)a	*0.0264*
Multiparity (≥ 2)	173 (86.93)a	110 (85.27)a	143 (64.71)b	*<0.0001*
Without partner	14 (7.07)a	19 (14.73)b	36 (16.36)b	*0.0120*
Nonwhite ethnicity	73 (36.87)	47 (37.01)	78 (35.29)	0.9270
Smoking	41 (19.81)	27 (20.30)	34 (14.91)	0.2995
Nonexercise/sedentarism	107 (54.04)a	86 (67.19)a	160 (74.42)b	< *0.0001*
Not working/housewife	116 (58.88)	72 (57.14)	106 (48.85)	0.0966
Family history of DM (1st degree)	95 (47.50)a	54 (41.54)a	59 (26.94)b	*< 0.0001*
Hypertension	75 (36.23)a	55 (41.67)ab	62 (27.43)a	*0.0161*
Prepregnancy BMI ≥ 25 kg/m^2^	169 (87.56)a	95 (74.80)b	124 (56.88)c	*< 0.0001*
Excessive weight gain	77 (42.54)	43 (37.72)	90 (43.48)	0.5885
OGTT-100 g (N = 220; mg/dL)^a^				
FPG ≥ 95	39 (54.93)a	6 (9.09)b	0 (0.00)b	*<0.0001*
1 h ≥ 180	50 (70.42)a	3 (4.55)b	1 (1.20)b	< *0.0001*
2 h ≥ 165	52 (73.24)a	4 (6.06)b	2 (2.41)b	< *0.0001*
3 h ≥ 140	41 (57.75)a	4 (6.06)b	0 (0.00)b	< *0.0001*
OGTT-75 g (N = 333; mg/dL)^b^				
FPG ≥ 92	77 (60.16)a	0 (0.00)b	0 (0.00)b	< *0.0001*
1 h ≥ 180	64 (51.20)a	0 (0.00)b	0 (0.00)b	< *0.0001*
2 h ≥ 153	63 (50.00)a	0 (0.00)b	0 (0.00)b	< *0.0001*
GP mean ≥ 120 mg/dL (at diagnosis)	14 (6.97)a	2 (1.54)b	1 (0.44)b	*0.0003*
HbA1c ≥ 6.5% (at birth)	21 (10.40)a	7 (5.51)a	2 (0.97)b	*0.0002*
HbA1c ≥ 6.0% (at birth)	57 (28.22)a	27 (21.26)a	15 (7.28)b	< *0.0001*

*GDM* gestational diabetes mellitus, *MGH* mild gestational hyperglycemia, *ND* nondiabetes, *DM* diabetes mellitus, *BMI* body mass index, *OGTT* oral glucose tolerance test, *FPG* fasting plasma glucose, *1* *h* 1-h postload, *2* *h* 2-h postload, *3* *h* 3-h postload, *GP* glucose profile, *HbA1c* glycate hemoglobin

* Chi squared or Fisher’s exact tests; for each specific variable, values followed by the same letter (a, b or c) are not significantly different (*p* ≥ 0.05); significant values were highlighted in italic

^a^Missed = 6; ^b^Missed = 10

**Table 2:** Perinatal outcomes according to glucose status

Perinatal outcomes	GDM	MGH	ND	*p*-value*
	N (%)	N (%)	N (%)	
Male	107 (51.69)	75 (56.39)	119 (51.97)	0.6530
C-section	140 (67.63)	89 (66.92)	132 (57.64)	0.0614
First C-section	52 (37.14)a	34 (38.20)b	76 (57.58)c	*0.0014*
GA < 37 weeks	20 (9.66)	5 (3.76)	14 (6.11)	0.0931
Apgar score 5th min < 7	7 (3.38)	3 (2.27)	9 (4.04)	0.6740
Apgar score 10th min < 7	1 (0.48)	0 (0.00)	1 (0.45)	0.7352
Birthweight (g)/GA				*0.0007*
SGA	12 (5.83)	13 (10.16)	33 (14.54)	
AGA	174 (84.47)	93 (72.66)	180 (79.30)	
LGA	20 (9.71)a	22 (17.19)b	14 (6.17)a	
Macrosomia	17 (8.21)a	19 (14.39)b	13 (5.68)a	*0.0171*
Cephal/Abdom < 1.0	5 (2.45)	4 (3.13)	7 (3.10)	0.9047
Ponderal index ≥ 2.98	62 (30.10)	50 (38.76)	60 (26.55)	0.0548
BW/GA Z-score > 2.0	9 (4.37)a	7 (5.43)a	1 (0.44)b	*0.0115*
Placental index > 0.21	38 (19.29)	18 (14.63)	32 (15.61)	0.4733
Malformation	5 (2.53)	5 (3.97)	6 (2.84)	0.7488
Umbilical cord blood				
Hematocrit > 65.0%	4 (2.16)	3 (2.75)	0 (0.00)	0.0810
Hematocrit > 55.0%	36 (19.46)a	12 (11.01)b	18 (8.96)b	*0.0073*
Bilirubin > 4.0 mg/dL	8 (4.21)	3 (2.61)	7 (3.37)	0.7542
Resuscitation at birth	39 (18.93)	21 (16.03)	38 (16.74)	0.7496
Respiratory distress^a^	15 (7.69)	9 (7.44)	13 (6.53)	0.8986
Phototherapy	54 (27.14)	30 (24.39)	52 (25.49)	0.8513
Sepsis	1 (0.51)	0 (0.00)	0 (0.00)	0.4395
NICU	6 (2.97)	0 (0.00)	7 (3.23)	0.1330
Perinatal death	0 (0.00)	1 (0.82)	1 (0.51)	0.4908
Hospital stay ≥ 4 days	55 (27.92)	25 (20.83)	54 (25.23)	0.3708
Any APO^b^	62 (29.9)	50 (37.6)	60 (26.2)	0.07465

*GDM* gestational diabetes mellitus, *MGH* mild gestational hyperglycemia, *ND* nondiabetes, *GA* gestational age, *SGA* small for gestational age, *AGA* adequate for gestational age, *LGA* large for gestational age, *Cephal/Abdom* cephalic/abdominal perimeter ratio, *BW* birthweight, *NICU* neonatal intensive care unit

^a^Respiratory distress—respiratory distress syndrome or meconium aspiration syndrome or persistent pulmonary hypertension or transient tachypnoea of the newborn

^b^Any adverse perinatal outcome (APO)—any of the following: preterm delivery, LGA, macrosomia, ponderal index ≥ 2.98, Apgar 5th min < 7, malformation, hospital stay ≥ 4 days or perinatal death [fetal OR neonatal until the 28th day]

* Chi squared or Fisher’s exact tests; for each specific variable, values followed by the same letter (a, b or c) are not significantly different (*p* ≥ 0.05); significant values are highlighted in italic

The frequency of the first C-section was lowest in the GDM group (37.14%), intermediate in the MGH group (38.20%) and highest in the control group (57.58%). The frequency of LGA newborns (GDM = 9.71%, MGH = 17.19%, control = 6.17%; p = 0.0007) and macrosomia (GDM = 8.21%, MGH = 14.39%, control = 5.68%; p = 0.0171) in the MGH group was higher than that observed in the GDM and control groups, both of which were statistically similar (MGH > GDM = control). The prevalence of Z-scores BW/GA > 2.0 was 4.37% in GDM, statistically similar to that in the MGH group (5.43%) and higher than that in the control group (0.44%) (*p* = 0.0115; GDM = MGH < control). A hematocrit level > 55.0% was observed in 19.46% of the GDM group, 11.01% of the MGH group and 8.96% of the control group (*p* = 0.0073; GDM > MGH = control) (Table 2).

Tables 3 and 4 show maternal and pregnancy characteristics and perinatal outcomes that are associated with HIP, comprising women in the GDM and MGH groups. Among those women, the presence of HIP was associated with age ≥ 25 years [1.81, 1.44–2.27], number of prenatal visits < 6 [1.33, 1.11–1.60], multiparity (pregnancy ≥ 2) [1.82, 1.43–2.31], nonexercise/sedentarism [0.77, 0.68–0.88], not working [1.17, 1.01–1.34], family history of DM [1.35, 1.18–1.54], hypertension [1.21, 1.06–1.38], and prepregnancy BMI ≥ 25 kg/m^2^ [1.82, 1.47–2.27]. As a glucose control marker, an HbA1c level ≥ 6.5 [1.57, 1.39–1.76] or ≥ 6.0% [1.51, 1.34–1.70] was also associated with HIP (Table 3). The perinatal outcomes associated with HIP were C-section [1.19, 1.02–1.38], LGA newborn [1.30, 1.10–1.54], macrosomia [1.26, 1.05–1.51], BW/GA Z-score > 2.0 [1.61, 1.40–1.84], hematocrit > 65.0% [1.70, 1.58–1.83] or > 55.0% [1.27, 1.07–1.50], and sepsis [1.63, 1.52–1.75]. Otherwise, the first C-section was less prevalent in the HIP group [0.74, 0.62–0.86] (Table 4).

**Table 3:** Maternal and pregnancy characteristics according to the study groups, with respective unadjusted risk ratios (RRs) and 95% confidence intervals (CIs)

Characteristics	HIP	Control	RR	Missing
	N (%)	N (%)	[95% CI]*	
Age ≥ 25 years	289 (85.25)	143 (62.72)	*1.81 [1.44–2.27]*	2
Prenatal visits < 6	34 (11.60)	11 (5.26)	*1.33 [1.11–1.60]*	67
Multiparity (≥ 2)	283 (86.28)	143 (64.71)	*1.82 [1.43–2.31]*	20
Without partner	33 (10.09)	36 (16.36)	0.78 [0.60–1.00]	22
Nonwhite ethnicity	120 (36.92)	78 (35.29)	1.03 [0.89–1.19]	23
Smoking	68 (20.00)	34 (14.91)	1.14 [0.98–1.34]	1
Nonexercise/sedentarism	193 (59.20)	160 (74.42)	*0.77 [0.68–0.88]*	28
Not working/housewife	188 (58.20)	106 (48.85)	*1.17 [1.01–1.34]*	29
Family history of DM (1st degree)	149 (45.15)	59 (26.94)	*1.35 [1.18–1.54]*	20
Hypertension	130 (38.35)	(27.43)	*1.21 [1.06–1.38]*	4
Prepregnancy BMI ≥ 25 kg/m^2^	264 (82.50)	124 (56.88)	*1.82 [1.47–2.27]*	31
Excessive weight gain	120 (40.68)	90 (43.48)	0.95 [0.82–1.11]	67
OGTT-100 g (N = 220; mg/dL)				
FPG ≥ 95	45 (32.85)	0 (0.00)	*1.90 [1.65–2.19]*	–
1 h ≥ 180	53 (38.69)	1 (1.20)	*1.94 [1.66–2.26]*	–
2 h ≥ 165	56 (40.88)	2 (2.41)	*1.93 [1.64–2.17]*	–
3 h ≥ 140	45 (32.85)	0 (0.00)	*1.90 [1.65–2.19]*	–
OGTT-75 g (N = 333; mg/dL)				
FPG ≥ 92	77 (40.53)	0 (0.00)	*2.23 [1.94–2.56]*	4
1 h ≥ 180	64 (34.22)	0 (0.00)	*2.13 [1.87–2.42]*	7
2 h ≥ 153	63 (33.51)	0 (0.00)	*2.11 [1.86–2.40]*	6
GP mean ≥ 120 mg/dL (at diagnosis)	16 (4.83)	1 (0.44)	*1.61 [1.40–1.85]*	13
HbA1c ≥ 6.5% (at birth)	28 (8.51)	2 (0.97)	*1.57 [1.39–1.76]*	34
HbA1c ≥ 6.0% (at birth)	34 (25.53)	15 (7.28)	*1.51 [1.34–1.70]*	34

*HIP* hyperglycemia in pregnancy (GDM + MGH), *DM* diabetes mellitus, *BMI* body mass index; *OGTT* oral glucose tolerance test, *FPG* fasting plasma glucose, *1* *h*: 1-h postload, *2* *h* 2-h postload, 3 h: 3-h postload, *GP* glucose profile, *HbA1c* glycate hemoglobin

* Significant values of RR [95% CI] are highlighted in italic

**Table 4:** Perinatal outcomes according to the study groups, with respective unadjusted risk ratios (RRs) and 95% confidence intervals (CIs)

Perinatal outcomes	HIP	Control	RR	Missing
	N (%)	N (%)	[95% CI]	
Male	182 (53.53)	119 (51.97)	1.03 [0.90–1.17]	–
C-section	229 (67.35)	132 (57.64	*1.19 [1.02–1.38]*	–
First C-section	86 (38.91)	76 (58.91)	*0.74 [0.62–0.86]*	–
GA < 37 weeks	25 (7.35)	14 (6.11)	1.08 [0.84–1.38]	–
Apgar score 5th min < 7	10 (2.95)	9 (4.04)	0.89 [0.56–1.34]	7
Apgar score 10th min < 7	1 (0.30)	1 (0.45)	0.83 [0.21–3.32]	8
LGA	42 (12.57)	14 (6.17)	*1.30 [1.10–1.54]*	8
Macrosomia	36 (10.62)	13 (5.68)	*1.26 [1.05–1.51]*	1
Cephal/Abdom < 1.0	9 (2.71)	7 (3.10)	0.94 [0.61–1.46]	11
Ponderal index ≥ 2.98	112 (33.43)	60 (26.55)	1.14 [0.99–1.31]	8
BW/GA Z-score > 2.0	16 (4.78)	1 (0.44)	*1.61 [1.40–1.84]*	8
Placental index > 0.21	56 (17.50)	32 (15.61)	1.05 [0.88–1.26]	44
Malformation	10 (3.09)	6 (2.84)	1.03 [0.70–1.52]	34
Hematocrit > 65.0%	7 (2.38)	0 (0.00)	*1.70 [1.58–1.83]*	74
Hematocrit > 55.0%	48 (16.33)	18 (8.96)	*1.27 [1.07–1.50]*	74
Bilirubin > 4.0 mg/dL	11 (3.61)	7 (3.37)	1.03 [0.71–1.50]	56
Resuscitation at birth	60 (17.80)	38 (16.74)	1.03 [0.87–1.23]	5
Respiratory distress^a^	24 (7.59)	13 (6.53)	1.06 [0.83–1.36]	54
Phototherapy	84 (26.09)	52 (25.49)	1.01 [0.87–1.18]	43
Sepsis	1 (0.32)	0 (0.00)	*1.63 [1.52–1.75]*	54
NICU	6 (1.83)	7 (3.2)	0.76 [0.42–1.38]	24
Perinatal death	1 (0.31)	1 (0.51)	0.81 [0.20–3.25]	53
Hospital stay ≥ 4 days	80 (25.24)	54 (25.23)	1.00 [0.85–1.17]	38
Any APO^b^	112 (32.94)	60 (26.20)	1.13 [1.00–1.30]	–

*HIP* hyperglycemia in pregnancy (GDM + MGH), *GA* gestational age, *SGA* small for gestational age, *AGA* adequate for gestational age, *LGA* large for gestational age, *Cephal/Abdom* cephalic/abdominal ratio, *BW* birthweight, *NICU* neonatal intensive care unit

^a^Respiratory distress—respiratory distress syndrome or meconium aspiration syndrome or persistent pulmonary hypertension or transient tachypnoea of the newborn

^b^Any APO—any of the following: preterm delivery, LGA, macrosomia, ponderal index ≥ 2.98, Apgar 5th min < 7, malformation, hospital stay ≥ 4 days or perinatal death [fetal OR neonatal until the 28th day]

In Table 5, the logistic regression analysis confirmed that maternal age ≥ 25 years [1.83, 1.12–2.99], prepregnancy BMI ≥ 25 kg/m^2^ [2.88, 1.89–4.39], family history of DM [2.12, 1.42–3.17] and multiparity [2.07, 1.27–3.37] were independent risk factors for HIP. Family history of DM [169, 1.16–2.16] and hypertension [2.00, 1.36–2.98] were independently associated with C-section. HbA1c ≥ 6.0% at birth was an independent risk factor for fetal overgrowth, which was indicated by LGA [1.99, 1.05–3.80], macrosomia [2.43, 1.27–4.63], and BW/GA Z-score > 2.0 [4.17, 1.57–11.10] (Table 5).

**Table 5:** Results of the logistic regression analysis—adjusted risk (RR_adj_) and 95% confidence intervals (CIs) of maternal and pregnancy characteristics (exposure) for HIP and perinatal outcomes (response)

Exposure	Outcome	RR_adj_	95% CI
Age ≥ 25 years	HIP	1.83	1.12–2.99
Prepregnancy BMI ≥ 25 kg/m^2^		2.88	1.89–4.39
Family history of DM (1st degree)		2.12	1.42–3.17
Multiparity (≥ 2)		2.07	1.27–3.37
Family history of DM (1st degree)	C-section	1.69	1.16–2.16
Hypertension		2.00	1.36–298
Prepregnancy BMI ≥ 25 kg/m^2^	1st C-section	0.45	0.27–0.57
HbA1c > 6.0%	LGA	1.99	1.05–3.80
	Macrosomia	2.43	1.27–4.63
	BW/GA Z-score > 2.0	4.17	1.57–11.10

Variables tested in the forward model: exposure = all significant results in Table 3 (maternal and pregnancy characteristics); outcomes = HIP (hyperglycemia in pregnancy) and all significant results in Table 4 (perinatal outcomes)

*HIP* hyperglycemia in pregnancy (GDM + MGH), *GA* gestational age, *LGA* large for gestational age, *BW* birthweight, *BMI* body mass index, *DM* diabetes mellitus, *HbA1c* glycate hemoglobin

Perinatal death occurred in two cases. Briefly, one was an early neonatal death in the nondiabetic group that occurred after term vaginal birth (Apgar 1st and 5th min = zero and Apgar 10th min = 2); the death was associated with maternal obesity and due to intrapartum hypoxia. The cause of the other perinatal death was unknown; the mother was in the MGH group and was severely obese; the newborn was AGA at term; the death also occurred after vaginal birth.

## Discussion

### Maternal characteristics and perinatal outcomes among different glucose statuses

The results of 569 pregnant women included in a Brazilian cohort of a public referral center showed similarities and differences in maternal and pregnancy characteristics dependent on glucose status. Such differences should be understood and discussed in light of diagnostic criteria and risk factors.

In our cohort, the 100 g- or 75 g-OGTT diagnostic tests were able to distinguish GDM, but not MGH, from control cases. The mean GP was also not an adequate test to identify either GDM or MGH. However, in a similar population, FPG ≥ 90 mg/dL and/or any postprandial level ≥ 130 mg/dL as measured by the GP test combined with normal 75 g-OGTT was able to identify MGH pregnant women (17.3%) [11]. Thus, our data reinforce previous considerations about the impact of the diagnostic protocol on the prevalence of hyperglycemia in pregnancy [11]. Besides, point out that the ideal diagnostic tests, and their respective cutoff points, remain undetermined for identifying women who would benefit from strict glucose control [26, 27].

As expected, the elevated HbA1c levels in late pregnancy differentiated both GDM and MGH from controls and can be used to assess the quality of glucose control in pregnancies complicated by hyperglycemia [20, 21]. According to recent studies, HbA1c values cannot replace OGTTs for the diagnosis of GDM, but it might be a useful tool to reduce the number of OGTTs, associated costs and the level of inconvenience to women [28, 29].

Both GDM and MGH pregnant women were older, multiparous, were more likely to have a family history of DM and were more adherent to the exercise practice. For this population, maternal age ≥ 25 kg/m^2^, parity ≥ 2, family history of DM and regular exercise were characteristics common to both MGH and GDM and represented a potential risk for hyperglycemia in pregnancy. Conversely, GDM was less frequent in pregnant women without partners.

Although controversial, maternal age ≥ 25 years, BMI ≥ 25 or 30 kg/m^2^, previous macrosomia and GDM, and a family history of DM were related to hyperglycemia in pregnancy [13, 17] and reinforce our results. However, it is surprising that regular exercise represents a risk for hyperglycemia in our cohort. This result may be explained by the higher prevalence of BMI ≥ 25 kg/m^2^ in the GDM and MGH groups and by our clinical protocol, which recommends exercise practice for all overweight or obese pregnant women, even before maternal hyperglycemia screening.

Regardless of the strict glucose control in the GDM and MGH groups [20, 21], adverse perinatal outcomes were more frequent in the MGH status group. The prevalences of first C-section, LGA, and macrosomia, which are direct or indirect markers of fetal overgrowth, were higher in the MGH group than in the control or GDM groups. These results point out two important issues. First, pregnant women in the GDM group were subjected to adequate glycemic control and obtained similar perinatal results to the control group (without hyperglycemia), and this is a good and expected result. Second, pregnant women with MGH had the worst perinatal results, which could not be expected.

Compared to the GDM group, the MGH group was less likely to have pregestational BMI ≥ 25 kg/m^2^, had a similar prevalence of excessive weight gain during pregnancy, and had adequate levels of HbA1c at birth, which could not prove the cause-effect relationship between MGH and fetal overgrowth. However, our pioneering study showed that the glucose profile showed a greater sensitivity and positive predictive value (PPV) for predicting fetal macrosomia, independent of the normal OGTT [9]. These results highlight the validity of the search for and the need for glucose control in patients with MGH [8–11, 24, 30, 31].

### The independent risk factors for HIP

To assess the risk factors for HIP, GDM and MGH were approached as a unique group, named hyperglycemia in pregnancy (HIP). After bivariate analysis, maternal age ≥ 25 years, prenatal visits < 6, multiparity, not working, family history of DM, hypertension, prepregnancy BMI ≥ 25 kg/m^2^, and elevated HbA1c levels at birth were associated with HIP; women who did not comply with the exercise recommendation were less prevalent in the HIP group. The logistic regression analysis confirmed that age ≥ 25 years, prepregnancy BMI ≥ 25 kg/m^2^, family history of DM and multiparity were independent risk factors for HIP. Interestingly, as shown in Table 1, the proportion of these independent risks was statically similar between the GDM and MGH groups. This finding reinforces the validity of evaluating GDM and MGH as a unique hyperglycemic condition.

For clinical practice, our results reinforce the use selective screening, in which only multiparous women with a family history of DM, aged ≥ 25 years, and BMI ≥ 25 kg/m^2^ would be directed to diagnostic tests (OGTT and GP). There is still debate in the literature about this topic [23–25]. While some authors indicate that a simple offer of an OGTT to women aged ≥ 25 years old and/or with a BMI ≥ 25 kg/m^2^ is as effective as more complex risk prediction models [7, 17], others concluded that risk-based screening may miss up to 30% of women with GDM because not all women have identifiable risk factors [13, 14, 16, 17, 26, 27, 32–34]. Our results may contribute to this issue, especially in populations and referral centers with similar characteristics.

Overweight and obesity is a real worldwide public health problem, as observed in our referral center. Previous studies have already identified the association between maternal adiposity and hyperglycemia, pointing out maternal age as the modulating factor in both multiparous [24, 35–39] and nulliparous women [40–42]. Although not fully defined, the current literature supports our findings.

### The independent risk factors for HIP-related adverse outcomes

In the present study, perinatal outcomes were worse in pregnancies complicated by HIP, independent of GDM or MGH. C-section, LGA and macrosomic newborns, Z-score BW/GA > 2.0, hematocrit levels > 65 or 55%, and sepsis were statistically associated with the HIP group. Conversely, the first C-section was more commonly associated with the control group, and fetal sex was not associated with HIP.

Specifically, for GDM, fetal and neonatal complications include C-section, macrosomia, shoulder dystocia, birth trauma, neonatal hypoglycemia and hyperbilirubinemia, and respiratory distress syndrome (RDS) [26, 43, 44]. In addition, milder forms, which do not fully meet GDM diagnostic criteria, were associated with hyperglycemia and adverse outcomes, including LGA, macrosomia, first C-section, and hospital length above 3 days [8–11]. Regardless of the inconsistency in perinatal outcomes evaluated in reviews or even in randomized trials [45], the literature supports our findings.

Some authors have reported increased rates of C-section and differences in outcomes relative to fetal sex, with a worse outcome for male newborns in GDM pregnancies [46, 47]. Our results are not consistent with these previous findings, probably due to the specificity of the population. In our cohort, the rates of first C-section were higher in the control group, thus potentially explaining the recurrence in this group and the lower rates in the HIP group. Relative to the influence of sex, the proportions of male and female newborns were equivalent, which may have contributed to the similarity in HIP outcomes.

The logistic regression analysis defined the independent risk factors for HIP adverse outcome. Family history of DM and hypertensive disorders were independently associated with C-section, and an HbA1c level > 6.0% was an independent risk factor for LGA, macrosomia, and Z-score BW/GA > 2.0. In contrast, prepregnancy BMI ≥ 25 kg/m^2^ was a protective factor for the first C-section.

Although the adverse outcomes evaluated in this study were associated with HIP, the logistic regression analysis did not identify the diagnostic tests and their cutoff points as independent risk factors. We also assessed maternal and pregnancy characteristics among DMG, MGH, and nondiabetic status. It is possible that the inadequacy of the tests and the fact that their cutoff points have yet to be defined could explain this issue [26, 27, 44]. HbA1c levels > 6.0% at late gestation were independently associated with fetal overgrowth as indicated by LGA, macrosomia, and Z-score BW/GA > 2.0. This result highlights the validity of strict glucose control [20, 21]. The HbA1c levels recommended for achieving glucose control during pregnancy are 6.0 to 6.5% [23]. According to our results, an HbA1c of 6.5% may be inadequate to prevent large or macrosomic newborns, and this issue must be confirmed in future studies.

In our study, a prepregnancy BMI ≥ 25 kg/m^2^ was confirmed as a protective factor against first C-section. As previously commented, this result may be population dependent. The higher first C-section rate in the control group and the management protocol of all overweight or obese pregnant women with nutritional counseling and exercise to prevent HIP could explain this question. Although not expected, this result highlights the potential benefit of lifestyle changes to prevent maternal hyperglycemia and its adverse outcomes [23, 36, 38].

Finally, our results support the association between maternal adiposity and hyperglycemia, and the maternal age as the modulating factor [24, 35–42]. Overweight and obesity appear to be the main drivers in HIP development. Thus, efforts must be made to improve optimal lifestyle management in childhood, adolescence, and adult life, particularly in the pre- and pregnancy phases, to curb the current epidemic of obesity due to its adverse repercussions for both mothers and newborns.

### Strength and limitations

Our study has some limitations. The sample size was calculated based on the frequency of LGA newborns and may compromise the statistical power for other perinatal outcomes evaluated. In this context, the specific characteristics of our referral center may make it difficult to reproduce the results. The strength of this study is that it includes different glucose statuses identified by 100 g- or 75 g-OGTT and glucose profiles, includes a sufficient sample size from a unique referral center, and uses well-defined diagnostic and management protocols, thus strengthen the consistency and quality of the data. In addition, our results raised important issues: (i) the validity of glucose control in MGH status and the need to detect and treat MGH in pregnant women; (ii) the possible inadequacy of the HbA1c cutoff at 6.5% to detect large or macrosomic newborns; and (iii) the potential benefit of lifestyle changes, with adequate diet and regular exercise, in the prevention of maternal hyperglycemia and its adverse outcomes. These issues lead to several possibilities for future research.

## Conclusions

The results of a Brazilian cohort referral center indicated that the intensity of maternal hyperglycemia affects pregnancy outcomes. MGH presents adverse pregnancy outcomes similar to those observed in the GDM group but distinct from the control (no diabetes) group. In our cohort, age ≥ 25 years, prepregnancy BMI ≥ 25 kg/m^2^, family history of DM, and multiparity were independent risk factors for HIP, supporting the selective screening for this condition.

Our results should be validated in populations with the same characteristics in Brazil or other low- or middle-income countries. Such results would provide evidence to determine the best approach for HIP diagnosis.

## Data Availability

All authors declare that data and any supporting material regarding this manuscript are available and can be requested at any time.
